# Telehealth Evaluation in the United States: Protocol for a Scoping Review

**DOI:** 10.2196/55209

**Published:** 2024-03-28

**Authors:** Yunxi Zhang, Yueh-Yun Lin, Lincy S Lal, Jennifer C Reneker, Elizabeth G Hinton, Saurabh Chandra, J Michael Swint

**Affiliations:** 1 Department of Data Science John D Bower School of Population Health University of Mississippi Medical Center Jackson, MS United States; 2 Center for Telehealth University of Mississippi Medical Center Jackson, MS United States; 3 Department of Management, Policy and Community Health The University of Texas School of Public Health Houston, TX United States; 4 Department of Population Health Sciences John D Bower School of Population Health University of Mississippi Medical Center Jackson, MS United States; 5 Rowland Medical Library University of Mississippi Medical Center Jackson, MS United States; 6 Department of Medicine University of Mississippi Medical Center Jackson, MS United States; 7 Institute for Clinical Research and Learning Healthcare John P and Katherine G McGovern Medical School The University of Texas Health Science Center at Houston Houston, TX United States

**Keywords:** cost, effectiveness, evaluation, framework, healthcare delivery, measurement, quality, scoping review, telehealth, United States

## Abstract

**Background:**

The rapid expansion of telehealth services, driven by the COVID-19 pandemic, necessitates systematic evaluation to guarantee the quality, effectiveness, and cost-effectiveness of telehealth services and programs in the United States. While numerous evaluation frameworks have emerged, crafted by various stakeholders, their comprehensiveness is limited, and the overall state of telehealth evaluation remains unclear.

**Objective:**

The overarching goal of this scoping review is to create a comprehensive overview of telehealth evaluation, incorporating perspectives from multiple stakeholder categories. Specifically, we aim to (1) map the existing landscape of telehealth evaluation, (2) identify key concepts for evaluation, (3) synthesize existing evaluation frameworks, and (4) identify measurements and assessments considered in the United States.

**Methods:**

We will conduct this scoping review in accordance with the Joanna Briggs Institute (JBI) methodology for scoping reviews and in line with the PRISMA-ScR (Preferred Reporting Items for Systematic Reviews and Meta-Analyses extension for Scoping Reviews). This scoping review will consider documents, including reviews, reports, and white papers, published since January 1, 2019. It will focus on evaluation frameworks and associated measurements of telehealth services and programs in the US health care system, developed by telehealth stakeholders, professional organizations, and authoritative sources, excluding those developed by individual researchers, to collect data that reflect the collective expertise and consensus of experts within the respective professional group.

**Results:**

The data extracted from selected documents will be synthesized using tools such as tables and figures. Visual aids like Venn diagrams will be used to illustrate the relationships between the evaluation frameworks from various sources. A narrative summary will be crafted to further describe how the results align with the review objectives, facilitating a comprehensive overview of the findings. This scoping review is expected to conclude by August 2024.

**Conclusions:**

By addressing critical gaps in telehealth evaluation, this scoping review protocol lays the foundation for a comprehensive and multistakeholder assessment of telehealth services and programs. Its findings will inform policy makers, health care providers, researchers, and other stakeholders in advancing the quality, effectiveness, and cost-effectiveness of telehealth in the US health care system.

**Trial Registration:**

OSF Registries osf.io/aytus; https://osf.io/aytus

**International Registered Report Identifier (IRRID):**

DERR1-10.2196/55209

## Introduction

### Overview

Telehealth has witnessed a remarkable transformation in recent years, propelled by the COVID-19 pandemic, which has greatly accelerated its adoption and expansion. The imperative for social distancing and the need to minimize in-person contact have made telehealth a pivotal tool at the forefront of health care delivery, helping to ensure accessibility and continuity of health care. This transformation has been further facilitated by significant expansions in telehealth service coverage, as well as regulatory requirements for telehealth visits, by the Centers for Medicare and Medicaid Services (CMS) and other payers, enhancing accessibility and affordability for patients across the nation [1-5].

The rapid expansion of telehealth services underscores the necessity for comprehensive guidance and evaluation of telehealth programs to guarantee the quality and effectiveness of health care delivery. The World Health Organization (WHO) has published a series of operational guidelines and evaluation frameworks to facilitate its global strategy on digital health development [6-9].

Recognizing the importance of maintaining service quality in practice, professional groups and telehealth stakeholders across the United States have actively contributed to the development of clinical guidelines. For instance, the American Telemedicine Association (ATA) published practice guidelines to enhance the technical quality and reliability of telemental health services for children and adolescents [10]. The ATA’s practice guidelines for ocular telehealth-diabetic retinopathy were updated to their third edition in 2020 to incorporate new evidence and technologies [11]. Furthermore, the American Heart Association (AHA) has advocated using remote patient monitoring technologies to improve cardiovascular disease outcomes [12], highlighting the ever-expanding role of telehealth in quality health care. The American Nurses Association (ANA) updated its core principles on telehealth in 2019 to provide guidance for health care professionals in delivering quality care using health technologies [13]. Similarly, the National Association of Social Workers (NASW) published guidance on legal considerations for telemental health, promoting adherence to state and federal practice guidelines and payer contract agreements among social workers [14].

Concurrently, in response to the rapidly evolving landscape of telehealth services, researchers, professional groups, and organizations have crafted telehealth evaluation frameworks. These frameworks have been designed to guide and facilitate the assessment of specific dimensions of telehealth programs. For example, Zhang et al [15] designed a framework to guide the development and evaluation of sustainable telehealth programs. Curfman et al [16] developed an economic framework focusing on measuring the value of pediatric telehealth. Moreover, a consortium of experts from the Kaiser Permanente (KP) Institute for Health Policy, AcademyHealth, the ATA, and the Physician Insurers Association of America (PIAA) collaborated on a telehealth research and policy framework, facilitating the assessment of health services and the quality of health care [17]. During the COVID-19 pandemic, the National Quality Forum (NQF) updated its telehealth measurement development framework, initially created in 2017, through an environmental scan conducted in 2021 [18-20].

Despite the wealth of telehealth evaluation frameworks available, several critical questions remain unanswered. First, the rapidly evolving state of telehealth programs, along with the emergence of innovative telehealth tools, has underscored the pressing need for a comprehensive grasp of the essential concepts that should be considered in their evaluation. For instance, the growing use of artificial intelligence in clinical assessments and the application of virtual reality among pediatric patients with autism spectrum disorder have garnered broad attention [21,22]. It is imperative to acquire a broader evaluation framework that accommodates these emerging technologies and provides a comprehensive understanding of integrating state-of-the-art technologies on telehealth platforms. As such, a scoping review that systematically identifies what telehealth services and programs are evaluated and how they are assessed is needed.

Additionally, while numerous frameworks have been proposed, many of them have been developed primarily from a narrow perspective or to address a specific need, potentially limiting their broader applicability and the comprehensiveness of the evaluation. For example, the framework developed by Zhang et al [15] focused on the sustainability of single telehealth programs, encompassing domains of program implementation, clinical effectiveness, and economic analysis. In contrast, the framework developed by KP, AcademyHealth, the ATA, and the PIAA considered five domains, including (1) policy context, (2) payment policy, (3) delivery, (4) modality, and (5) outcomes [17]. In the case of the NQF telehealth measurement development framework, it emphasizes assessing the impact of telehealth on health care system readiness and health outcomes in rural areas, spanning across another five domains: (1) access to care and technology; (2) costs, business model, and logistics; (3) experience; (4) effectiveness; and (5) equity [15,18,19]. Given the diverse array of telehealth stakeholders in the United States, including patients, providers, hospitals, payers, professional associations, federal agencies, policy makers, and legislators, it is necessary to consider the perspectives of multiple stakeholders and comprehensively evaluate telehealth services and programs.

Moreover, the measurements and assessments associated with telehealth evaluation domains and frameworks remain unclear, raising questions about how to effectively gauge the impact and outcomes of telehealth services and programs. While the frameworks developed by the WHO, Zhang et al [15], and NQF outline the measurements to be considered, this clarity is absent in the frameworks developed by KP, AcademyHealth, the ATA, and the PIAA [6-9,17,18].

### Objective and Review Question

The broad objective of this scoping review is to answer the question of what is known about telehealth services and program evaluation in the United States. Specifically, this scoping review will be conducted to (1) map the existing landscape of telehealth evaluation, (2) identify key concepts for evaluation, (3) synthesize existing frameworks, and (4) identify measurements and assessments that have been considered.

A preliminary search of PROSPERO, MEDLINE, the Cochrane Database of Systematic Reviews, the Open Science Framework, and JBI Evidence Synthesis was conducted, and no current or in-progress scoping reviews or systematic reviews on the topic were identified.

## Methods

### Study Design

The scoping review will be conducted in accordance with the Joanna Briggs Institute (JBI) methodology for scoping reviews and in line with the PRISMA-ScR (Preferred Reporting Items for Systematic Reviews and Meta-Analyses extension for Scoping Reviews) [23-25].

### Protocol and Registration

The protocol for this scoping review is registered on the Open Science Framework (osf.io/aytus) [26]. The scoping review is expected to be completed in 6 months.

### Eligibility Criteria

The eligibility for this scoping review is elaborated following the Population, Concept, and Context (PCC) framework.

#### Participants

This scoping review will consider a range of document types, including reviews, reports, and white papers, specifically related to telehealth evaluation frameworks and associated measurements. The evaluation frameworks to be included will focus on telehealth services and programs used for the provision of health services through well-established modalities, such as store-and-forward telemedicine, remote monitoring, real-time counseling, audio and video conferencing, and videotelephony, as well as emerging innovations integrated with telehealth platforms, such as virtual realities and artificial intelligence [27]. We will exclude manuscripts with data analyses only, case studies, project intervention reports (including scale-up and scale-down studies), and commentaries.

#### Concept

This review will examine concepts pertaining to the evaluation frameworks of telehealth services and programs. The concepts to be examined will encompass existing evaluation frameworks and associated measurements developed by telehealth stakeholders, professional organizations, and authoritative sources, excluding those developed by individual researchers. This approach will allow us to collect data that reflect the collective expertise and consensus of experts within the respective professional group. The aim is to create a comprehensive overview of telehealth evaluation, incorporating perspectives from multiple stakeholder categories, including but not limited to public and private payers, providers, and policy makers.

#### Context

This review will consider documents published in English for various health care settings, for example, primary care, specialty care, and rural health care for adult and pediatric patients. Considering the diversity of the contexts of telehealth services and programs across different health care systems and the unique nature of the US health care system, publications reporting on evaluation frameworks developed for regions that do not include the United States will be excluded. Documents published by worldwide health organizations will be included, given their relevance and influence in US health care.

### Search Strategy

As many evaluation frameworks are likely to be published on stakeholders’ websites, we will source data from peer-reviewed journals and gray literature, such as reports, white papers, policy documents, and guidelines.

The search strategy will aim to locate published reviews, reports, and white papers. An initial PubMed search was undertaken to identify articles on the topic. The text words contained in the titles and abstracts of relevant articles and the index terms used to describe the articles were used to develop a full search strategy for PubMed (Multimedia Appendix 1). The search strategy, including all identified keywords and index terms, will be adapted for each included database and information source. The reference lists of all included studies will be screened for additional titles. The databases to be searched include PubMed (US National Library of Medicine), Health Technology Assessments (International Network of Agencies for Health Technology Assessment), and Web of Science Core Collection (Clarivate Analytics). The websites of telehealth stakeholders, professional organizations, and authoritative sources mentioned in the included articles will be screened for additional documents.

Documents published from January 1, 2019, to the present will be considered for inclusion. While we recognize the relevance of earlier publications, the rapid expansion of telehealth, particularly accelerated by the COVID-19 pandemic, has led to significant changes in telehealth services and programs. During and after the pandemic, not only did the volume of telehealth services increase, but there was also a diversification in the types of modalities considered under the telehealth umbrella. For example, before the pandemic, audio-only interactions were not widely regarded as telehealth, and various modalities like e-consults and e-visits were not as commonly used as they are now. Therefore, the inclusion of documents published from January 1, 2019 onward will allow us to focus on the most recent and relevant telehealth service and program evaluations.

### Source of Evidence Selection

Following the search, all identified records will be collated and uploaded into EndNote (V21; Clarivate Analytics), with duplicates removed. A total of 2 reviewers will independently screen titles and abstracts against the inclusion criteria. Following a pilot test, titles and abstracts will then be screened by 2 independent reviewers for assessment against the inclusion criteria for the review. All screening will be completed through Rayyan, a web-based tool for evidence synthesis projects [28]. The full text of selected citations will be assessed in detail against the inclusion criteria by 2 or more independent reviewers. Reasons for the exclusion of full-text documents that do not meet the inclusion criteria will be recorded and reported in the scoping review. Any disagreements that arise between the reviewers at each stage of the selection process will be resolved through discussion. The results of the search will be reported in full in the final scoping review and presented in a PRISMA (Preferred Reporting Items for Systematic Reviews and Meta-Analyses) flow diagram [29].

### Data Extraction

Data will be extracted from documents selected for inclusion in the scoping review by 2 independent reviewers using a data extraction tool developed by the reviewers based on the JBI data extraction template for scoping review [24]. The data extracted will include specific details about the PCC methods and key findings relevant to the review question. A draft extraction tool is provided in Multimedia Appendix 2. The draft data extraction tool will be modified and revised as necessary during the process of extracting data from each included document. Modifications will be detailed in the full scoping review. Any disagreements that arise between the reviewers will be resolved through discussion.

## Results

Reviewers will synthesize data across selected documents using tools such as tables and figures. Frequency counts of domains and measurements considered in frameworks and guidelines will be presented when applicable to highlight key patterns. Visual aids like Venn diagrams will be used to illustrate the relationships between the evaluation frameworks from various sources. Additionally, a narrative summary will be crafted to further describe how the results align with the review objectives, facilitating a comprehensive overview of the findings. This scoping review is expected to conclude by August 2024.

## Discussion

### Implications

In light of the rapid expansion of telehealth services, this scoping review seeks to address critical gaps in the current understanding of telehealth evaluation. The urgency of this endeavor is underscored by the need to ensure the quality, effectiveness, and cost-effectiveness of telehealth programs, with their increasing significance to the US health care system.

This scoping review will be the first to synthesize existing evidence of telehealth services and program evaluation in the United States and to facilitate the future development of telehealth in the postpandemic era. It will provide insights into the evaluation of state-of-the-art technology integration into telehealth.

In addition, the synthesized evaluation concepts and frameworks crafted by multiple telehealth stakeholders will guide the development of a multistakeholder evaluation framework that allows the comprehensive assessment of telehealth services and programs. Specifically, a multistakeholder framework will offer an inclusive and adaptable approach to telehealth evaluation, making it relevant and valuable to a wide range of users. By accommodating the diversity of telehealth initiatives, technologies, and objectives that exist within the US health care system, it will provide flexibility for stakeholders to tailor their evaluations for specific needs and objectives. In the evolving landscape of telehealth, where innovations and changes occur regularly, new technologies, practices, and policies would also be considered in the multistakeholder framework. The consideration of measurements and assessments will further illuminate the path toward actionable telehealth evaluation by providing more context and details.

### Limitations

While we aim to conduct a comprehensive scoping review, some limitations should be considered. First, to ensure the comprehensiveness and relevance of this study’s findings, we only consider frameworks developed for regions that encompass the United States. For instance, frameworks from organizations like the WHO are considered if they pertain to the US context. However, given the unique and complex nature of the US health care system, the results from this scoping review might not be directly applicable to other countries. In addition, given that the scoping review aims to provide a comprehensive overview of telehealth evaluation from a multistakeholder perspective, the scoping review will exclude documents carried out by individual researchers. Therefore, when evaluating local telehealth programs, it is advisable to include specific local considerations in addition to the results of this scoping review.

### Conclusions

This scoping review will serve as a critical initial step in advancing the understanding of telehealth evaluation, given the current role of telehealth in the US health care system. Its findings will inform policy makers, health care providers, researchers, and other stakeholders involved in the rapidly evolving field of telehealth. By addressing these fundamental questions, this protocol lays the foundation for the scoping review for comprehensive telehealth evaluation and future telehealth advancement.

## Appendix

Multimedia Appendix 1 Search strategy.

Multimedia Appendix 2 Data extraction instrument.

## Abbreviations

AHA: American Heart Association
ANA: American Nurses Association
ATA: American Telemedicine Association
CMS: Centers for Medicare and Medicaid Services
JBI: Joanna Briggs Institute
KP: Kaiser Permanente Institute for Health Policy
NASW: National Association of Social Workers
NQF: National Quality Forum
PCC: Population, Concept, and Context
PIAA: Physician Insurers Association of America
PRISMA: Preferred Reporting Items for Systematic Reviews and Meta-Analyses
PRISMA-ScR: Preferred Reporting Items for Systematic Reviews and Meta-Analyses extension for Scoping Reviews
WHO: World Health Organization
