# Supplementary material for: Telehealth Evaluation in the United States: Protocol for a Scoping Review
Source: JMIR Res Protoc. 2024 Mar 28;13:e55209. doi: 10.2196/55209 (PMC11009841; doi:10.2196/55209)
Supplement: Multimedia Appendix 1 [file resprot_v13i1e55209_app1.docx]

# Multimedia Appendix I: Search strategy

PubMed
Search conducted on November 22, 2023

| **Search** | **Query** | **Records retrieved** |
| --- | --- | --- |
| #1 | "Program Evaluation"[Mesh] OR evaluat*[tw] OR assess*[tw] | 7,820,763 |
| #2 | "Guideline" [Publication Type] OR "Guidelines as Topic"[Mesh] OR guideline[tiab] OR framework[tiab] | 643,494 |
| #3 | "Telemedicine"[Mesh] OR telemedicine[tiab] OR telecare[tiab] OR virtual medicine[tiab] OR telehealth[tiab] OR telenursing[tiab] OR telemonitoring[tiab] OR tele monitoring[tiab] OR telemetric[tiab] OR telehomecare[tiab] OR tele homecare[tiab] OR telemanagement[tiab] OR tele management[tiab] OR telerehabilitation[tiab] OR telerehabilitation[tiab] OR tele rehabilitation[tiab] OR telesurveillance[tiab] OR tele surveillance[tiab] OR remote patient monitoring[tiab] OR remote monitoring[tiab] OR remote care[tiab] OR "Remote Consultation"[Mesh] OR remote consultation[tiab] OR teleconsultation[tiab] OR ehealth[tiab] OR e-Health[tiab] OR mhealth[tiab] OR mobile health[tiab] OR m-Health[tiab] OR real-time counseling[tiab] OR "Videoconferencing"[Mesh] OR videoconferenc*[tiab] OR audio and video conferencing[tiab] OR videotelephony[tiab] OR digital health[tiab] OR virtual care[tiab] OR audio visit[tiab] OR tele[tw] OR (tele[tiab] AND point of care[tiab]) | 94,634 |
| #4 | 1 AND 2 AND 3 | 3,509 |
| Limited to English language and 2019—present | | 2,313 |
