# Supplementary material for: Telehealth Evaluation in the United States: Protocol for a Scoping Review
Source: JMIR Res Protoc. 2024 Mar 28;13:e55209. doi: 10.2196/55209 (PMC11009841; doi:10.2196/55209)
Supplement: Multimedia Appendix 2 [file resprot_v13i1e55209_app2.docx]

# Multimedia Appendix II: Data extraction instrument

| Author(s) | The last name(s) of author(s). E.g., Smith; Smith & Doe; Smith et al. |
| --- | --- |
| Title | Full title of the article. |
| Year | Publication year of the article. |
| Types of evidence source | This could be peer-reviewed journal article, professional group websites, or any other relevant sources. |
| Context | The telehealth evaluation context. E.g., U.S. or worldwide. |
| Aims/purpose | Summarize the aims and purpose of the article. |
| Population | The target population or groups that the of the telehealth evaluation focus on. This could include specific patient populations, healthcare professionals, or other relevant groups. |
| Healthcare setting | The healthcare setting of the evaluation context. This could be primary care, specialty care, rural healthcare, etc. |
| Telehealth modalities | List telehealth modalities or technologies applied in the framework. This might include store-and-forward telemedicine, remote monitoring, real-time counseling, audio and video conferencing, etc. |
| Framework | Summarize the proposed framework. |
| Domain | Identify the primary categories addressed by the framework. These are typically the first layer of the categories or themes that structure the framework, such as effectiveness. |
| Subdomain | If applicable, specify the subcategories within the primary categories. These are typically the second layer of the categories that further structure the identified domains. For example, within the effectiveness domain, subdomains might include clinical effectiveness. |
| Measurement | Quantitative functions or assessment metrics to describe the concepts within a domain or subdomain. This could include measurement tools, scales, and metrics applied to evaluate telehealth services and programs. |
